# Imaging Anatomy of the Ankle in Normal and Pathological States: A Clinically Focused Pictorial Review

**DOI:** 10.7759/cureus.93882

**Published:** 2025-10-05

**Authors:** Omar González-Gutiérrez, Ernesto Roldan-Valadez, Mauricio Molina-Gonzalez, Martin-Romo Garcia-de-Bustamante, Luis-Octavio López-Montoya, Andrea-Paola Gonzalez-Trejo, Jesica Alvarez-Hernández, Samaria Perez-Galindo

**Affiliations:** 1 Medicine, Universidad Cuauhtémoc Querétaro, Santiago de Querétaro, MEX; 2 Imaging Service, Centro Médico Jurica, Santiago de Querétaro, MEX; 3 Magnetic Resonance Unit, Diagnóstico Milenio, Santiago de Querétaro, MEX; 4 Research, Instituto Nacional de Rehabilitacion "Luis Guillermo Ibarra Ibarra", Mexico City, MEX; 5 Radiology, I.M. Sechenov First Moscow State Medical University, Moscow, RUS; 6 Research, Instituto Nacional de Rehabilitación "Luis Guillermo Ibarra Ibarra", Mexico City, MEX; 7 Imaging Service, Hospital General Regional No. 2 El Marqués, Instituto Mexicano del Seguro Social (IMSS), Santiago de Querétaro, MEX; 8 Orthopedics and Traumatology, Hospital General de Pachuca, Pachuca de Soto, MEX

**Keywords:** ankle imaging, ankle instability, computed tomography, imaging anatomy, ligament injury, magnetic resonance imaging, osteochondral lesion, pictorial review, tendon pathology, ultrasound

## Abstract

This narrative pictorial review presents the imaging anatomy of the ankle in normal and pathologic states and emphasizes practical, clinically oriented correlations. The ankle is a compound synovial joint complex in which osseous architecture, ligamentous systems (lateral collateral, deltoid, and syndesmotic), and tendon compartments interact within a compact space to provide stability and motion during gait and sport. This anatomical complexity underlies a broad spectrum of traumatic and degenerative conditions, including lateral ankle sprains, syndesmotic injury, osteochondral lesions of the talus, tendinopathy/rupture, and post-traumatic osteoarthritis, that are highly prevalent in clinical practice. Normal imaging anatomy provides the baseline for detecting disease: radiographs remain indispensable for initial assessment, ultrasound offers dynamic tendon-ligament evaluation, CT delineates osseous architecture and alignment, and MRI affords comprehensive soft-tissue and osteochondral characterization. Emerging techniques - weight-bearing CT and high-resolution isotropic or quantitative MRI, with MR arthrography when indicated - improve detection of subtle instability and early cartilage or capsuloligamentous injury. Evidence informing this review was derived from a non-systematic search of MEDLINE/PubMed, Embase, Scopus, and the Cochrane Library (January 1990-September 2025; last update 12 September 2025), prioritizing studies from the past two decades while retaining seminal references. By coupling descriptive imaging anatomy with representative traumatic and degenerative patterns, the review aims to support diagnostic accuracy, multidisciplinary communication, and evidence-based management of ankle disorders.

## Introduction and background

The ankle joint is a highly congruent articulation essential for bipedal stance, gait, and the absorption of mechanical forces during daily and athletic activity. Its anatomy integrates osseous, ligamentous, tendinous, muscular, and neurovascular structures within a compact space, providing both mobility and stability. This complexity also renders the ankle vulnerable to a wide range of conditions, including acute ligament sprains, tendon ruptures, osteochondral lesions, and post-traumatic osteoarthritis. These injuries are among the most common musculoskeletal problems in clinical practice and a leading cause of disability in young athletes and older adults alike [1].

Because the talar dome is wider anteriorly than posteriorly, dorsiflexion tightens the talocrural mortise, while plantarflexion relaxes it, making the ankle more dependent on ligamentous stability and thus more susceptible to inversion sprains. The lateral collateral ligament (LCL) complex is most frequently injured, especially the anterior talofibular ligament (ATFL) [2]. Chronic ankle instability develops in up to 40% of patients after a significant sprain [3].

Advances in imaging have greatly improved the evaluation of ankle anatomy and pathology. Conventional radiography remains the first-line modality for acute injuries, particularly to assess fractures and joint alignment. Musculoskeletal ultrasound provides dynamic, real-time visualization of superficial tendons and ligaments and has excellent sensitivity for chronic ATFL tears when performed by experienced operators [3]. Computed tomography (CT) is indispensable for complex fractures and preoperative planning, while magnetic resonance imaging (MRI) remains the gold standard for evaluating soft tissue, cartilage, and bone marrow pathology [4]. Magnetic resonance arthrography can increase diagnostic yield for subtle ligamentous and chondral injuries, particularly in chronic instability [5]. Newer methods, such as weight-bearing CT (WBCT) and 3D isotropic MRI, are increasingly used to detect subtle malalignments and evaluate cartilage load distribution.

Given this complexity, accurate interpretation of ankle imaging requires not only technical knowledge of modalities but also a solid understanding of normal anatomy, anatomical variants, and injury biomechanics. This review provides a clinically oriented overview of ankle anatomy, integrating advanced imaging techniques and arthroscopic findings to offer practical diagnostic tools and foster effective multidisciplinary communication in the management of ankle disorders.

## Review

Methods

This article was designed as a narrative review with the objective of integrating anatomical, biomechanical, and clinical knowledge of the ankle with imaging correlations relevant to everyday clinical practice. Therefore, as a narrative, clinically oriented pictorial review rather than a systematic or scoping review, a Preferred Reporting Items for Systematic Reviews and Meta-Analyses (PRISMA) flow diagram was not generated. To ensure transparency appropriate for a narrative review, we conducted a narrative literature search of MEDLINE/PubMed, Embase, Scopus, and the Cochrane Library covering January 1990 through September 2025; the last update was performed on 12 September 2025. Search strings combined controlled vocabulary and keywords (e.g., "ankle anatomy," "talocrural," "subtalar," "ligament*," "deltoid," "syndesmosis," "tendon*," "Achilles," "ultrasound," "MRI," "isotropic," "arthrography," "CT," "weight-bearing CT"). We applied filters for humans, the English language, and adolescents and adults. Eligible article types included anatomical studies, imaging studies, clinical series, reviews, guidelines, and consensus statements; we excluded single-patient case reports and conference abstracts unless they illustrated key imaging landmarks. To ensure currency, we prioritized publications from 2014 to 2025 while preserving seminal papers that define anatomy and imaging correlations [6-13]. Two reviewers (MGM, OGG) screened titles/abstracts independently and resolved disagreements by consensus.

The final selection of references aimed to balance classic anatomical descriptions with recent clinical and imaging advances (e.g., MRI traction techniques, WBCT, and 3D isotropic MRI). Figures and tables were created to illustrate ligamentous and tendinous structures, as well as their clinical and imaging relevance. This narrative pictorial did not apply formal risk-of-bias tools (e.g., Quality Assessment of Diagnostic Accuracy Studies, Version 2 (QUADAS-2) or Risk Of Bias In Non-randomized Studies of Interventions (ROBINS-I)) and did not perform quantitative synthesis or meta-analysis; consequently, no hypothesis testing, p-values, or confidence intervals were reported, and statistical peer review was not required for this design. The review aimed to integrate authoritative anatomical descriptions and contemporary imaging evidence to inform clinical decision-making in routine practice. Emphasis was placed on presenting a clinically oriented overview, highlighting correlations between normal anatomy, traumatic and degenerative pathologies, and the practical role of imaging in diagnosis and treatment planning.

For a concise modality-by-modality comparison tailored to clinical decision-making, Table 1 summarizes roles, strengths, limitations, and typical use cases across radiography, ultrasound, MRI (including isotropic 3D sequences), MR arthrography, CT, and WBCT [3,5,11,14-16].

**Table 1:** Comparative imaging modalities for the ankle: roles, strengths, limitations, and typical use cases. Summary of common modalities used in ankle assessment. WBCT: weight-bearing computed tomography; AP: anteroposterior; MRA: magnetic resonance arthrography; ATFL: anterior talofibular ligament; CFL: calcaneofibular ligament; PTFL: posterior talofibular ligament Credits: Original table created by the authors; not reproduced from previously published material; no third-party permissions required.

Modality	Primary role	Key strengths	Key limitations	Typical use cases/findings	Key references
Radiography (AP, lateral, mortise; stress when indicated)	First-line structural assessment	Widely available; alignment and joint spaces; fracture detection; stress views for instability	Limited soft-tissue contrast; subtle osteochondral lesions may be occult	Fracture/equipment triage; mortise integrity; syndesmotic and lateral clear space assessment	[11,15]
Ultrasound	Dynamic tendon/ligament evaluation	Real-time, dynamic; high spatial resolution for superficial structures; guides interventions	Operator dependent; limited deep/intra-articular assessment	Peroneal tendon split/subluxation; ATFL/CFL tears; Achilles tendinopathy; guided injections	[10,17]
MRI (2D)	Comprehensive soft-tissue and osteochondral evaluation	Multiplanar soft-tissue detail; marrow edema; cartilage/subchondral changes	Cost; availability; motion sensitivity	Ligament tears (ATFL/CFL/PTFL; deltoid); tendon pathology; osteochondral lesions; impingement	[5,7,18,19]
MRI (isotropic 3D)	Thin structure depiction and reformats	Isotropic voxels; high-detail multiplanar reconstructions	Longer scans; sequence availability varies	Fine capsuloligamentous anatomy; pre-op planning; subtle partial-thickness tears	[4,5]
MRA (direct)	Intra-articular detail and subtle tears	Distends recesses; improves detection of partial-thickness/intracapsular defects	Invasive; contrast contraindications	Subtle ATFL/deltoid deep fiber tears; anterolateral recess pathology; adhesions	[5,16,18,19]
CT (non-weight-bearing)	Cortical/osteophyte detail; osseous architecture	Excellent bone detail; rapid	Poor soft-tissue contrast; radiation	Fracture characterization; osteophyte mapping; pre-op planning	[15]
WBCT	Alignment under load; subtle diastasis	Functional assessment of mortise and hindfoot alignment	Availability; radiation; limited soft-tissue	Dynamic malalignment; subtle syndesmotic widening; pre-/post-operative comparison	[11,14]

Normal anatomy of the ankle

The ankle is one of the most congruent and therefore most stable joints of the lower limb. Functionally, the ankle is a compound synovial joint complex that combines hinge-type dorsiflexion-plantarflexion at the talocrural articulation with coupled inversion-eversion through the subtalar complex, all stabilized by robust capsuloligamentous structures [6,7,12,20]. The principal static stabilizers are grouped into three systems: the lateral collateral complex-anterior talofibular, calcaneofibular, and posterior talofibular ligaments (PTFLs); the medial collateral (deltoid) complex with superficial and deep layers; and the distal tibiofibular syndesmotic complex [6,9,21,22]. Together with dynamic support from the surrounding tendons, these ligaments resist inversion-eversion, translational, and rotational stresses that are common in sport-related injury [7,20,21].

Notably, the anterior capsular insertion is located approximately 4 mm proximal to the tibial articular surface and 2.5 mm proximal to the talar dome [13]. This creates an anterior recess that expands during dorsiflexion, a characteristic that facilitates arthroscopic access in clinical practice.

Articular components

**Figure 1:**
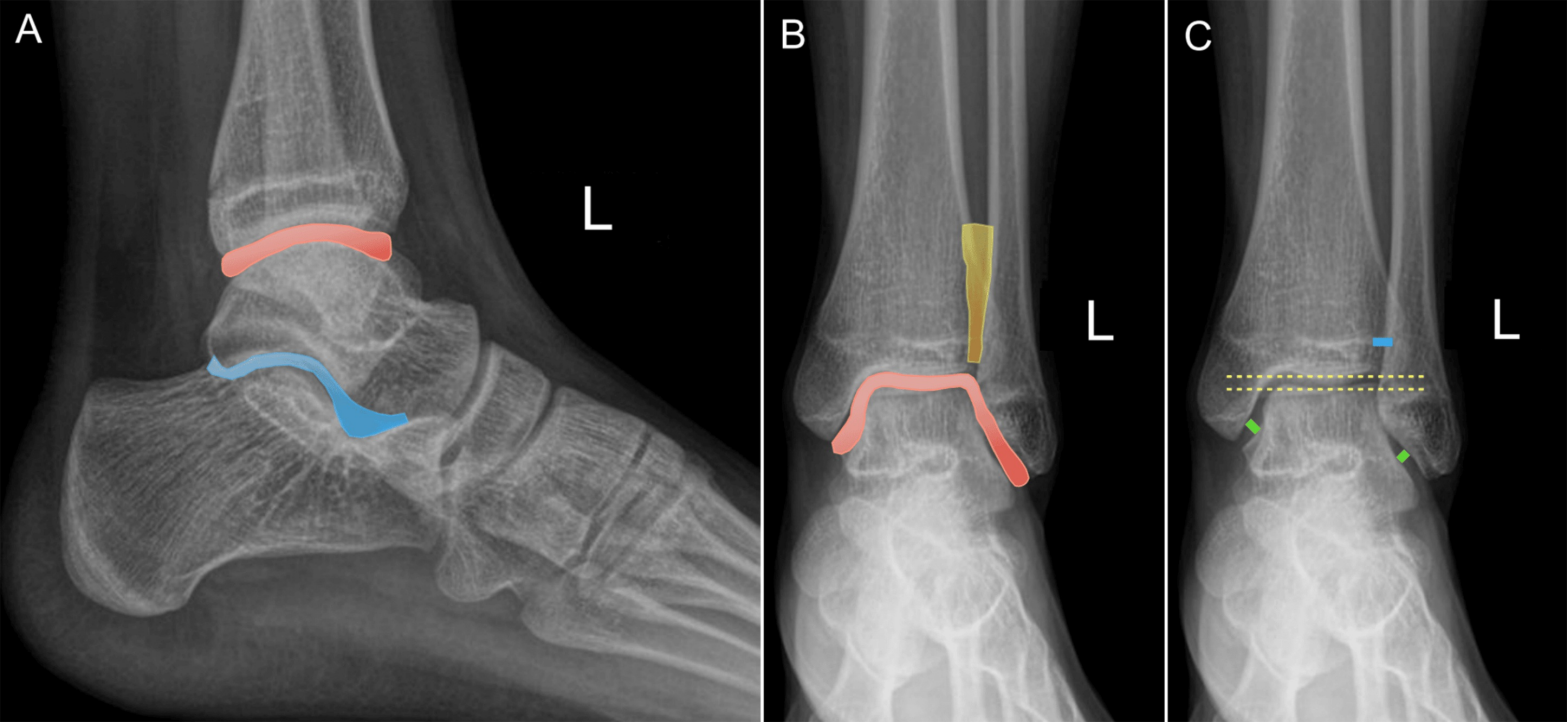
Normal ankle radiographs in a 16-year-old female. Anteroposterior and lateral radiographs demonstrate (A, B) the tibiotalar joint (red highlight), subtalar joint (blue), and syndesmotic joint (yellow); (C) normal tibio-talar parallelism (yellow dashed line), uniform joint space (green lines), and tibiofibular space (blue line). Images belong to the authors; patient consent was obtained for publication.

The osseous structures of the ankle joint include the tibia, fibula, talus, and calcaneus. Together, they form two proximal articulations - the talocrural and subtalar joints - and one distal fibrous articulation, the tibiofibular syndesmosis (Figures 1A, 1B). The talocrural joint allows a sagittal range of motion of approximately 13-33° in dorsiflexion and 23-56° in plantarflexion [6], while also permitting inversion and eversion movements.

The “roof” of the talocrural joint is formed by the tibial plafond and distal fibula, creating a concave quadrilateral surface with a central crest. Laterally, the articular surface is provided by the fibular malleolus, triangular with its apex directed caudally, and medially by the tibial malleolus. The talar dome forms the base of the articulation, with a pulley-shaped profile, a central groove, and two lateral ridges. The tibio-talar joint space should measure approximately 4 mm and remain uniform throughout its course [11].

In the neutral position, the tibial and talar surfaces should be parallel, a relationship known as tibio-talar parallelism (Figure 1C). Divergence greater than 5° on stress radiographs is considered abnormal and suggests ligamentous instability [11]. This measurement is frequently used in sports medicine and orthopedic settings to confirm lateral ankle sprains.

The distal tibiofibular syndesmosis is a fibrous joint that allows controlled axial, rotational, and translational motion, playing a critical role in distributing forces through the ankle mortise [12]. On radiographs, the tibiofibular clear space should measure less than 5 mm at 1 cm above the tibial plafond. A wider distance raises suspicion for syndesmotic injury, especially in the setting of high ankle sprains (Figure 1C).

Clinical relevance

Understanding the articular anatomy of the ankle is essential in both the acute and chronic clinical settings. Subtle misalignment of the talocrural joint or unrecognized syndesmotic widening can predispose patients to chronic instability, altered gait mechanics, and eventually post-traumatic osteoarthritis. Imaging evaluation of these anatomical structures, particularly using weight-bearing radiographs, CT for osseous congruity, and MRI for ligament integrity, plays a pivotal role in the accurate diagnosis and treatment planning of ankle trauma.

Ligamentous anatomy

The ligamentous support of the ankle is classically divided into three complexes: the LCL, the medial collateral ligament or deltoid complex, and the syndesmotic complex. Together, these structures provide passive stabilization against inversion, eversion, rotation, and translational forces, complementing the dynamic stability afforded by the surrounding musculotendinous system.

**Figure 2:**
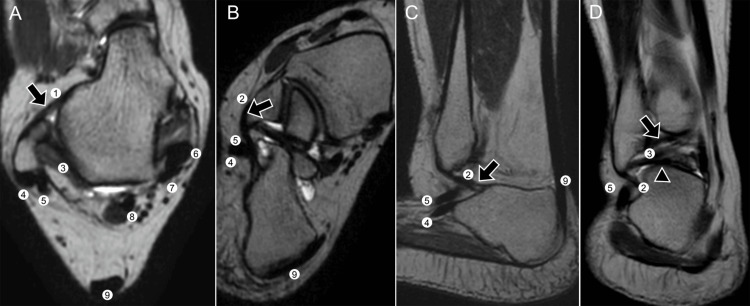
Lateral ligament complex on volumetric T2-weighted CUBE MRI. Multiplanar reconstructions depict (1) anterior talofibular ligament (ATFL), (2) calcaneofibular ligament (CFL), (3) posterior talofibular ligament (PTFL), (4) peroneus longus tendon, (5) peroneus brevis tendon, (6) posterior tibial tendon, (7) flexor digitorum longus tendon, (8) flexor hallucis longus (FHL) tendon, and (9) calcaneal (Achilles) tendon. Three-dimensional views show (A) ATFL (arrow); (B, C) CFL (arrow); (D) PTFL including deep fibers (arrow) and superficial fibers (arrowhead). Images belong to the authors; patient consent was obtained.

The LCL complex is composed of the ATFL, the calcaneofibular ligament (CFL), and the PTFL (Figure 2). Among these, the ATFL is the most frequently injured due to its relatively thin structure and the fact that it resists anterior talar translation and plantarflexion [7,20].

Anatomically, it originates at the anterior margin of the lateral malleolus and inserts on the lateral talar neck, and it may be composed of one to three distinct bands. The CFL originates from the anterior aspect of the lateral malleolus and courses obliquely in a posteroinferior direction to insert on the posterolateral calcaneus. Measuring approximately 20 mm in length and 6-8 mm in diameter, it is unique among ankle ligaments in stabilizing both the talocrural and subtalar joints simultaneously [21]. The PTFL is the strongest of the lateral ligaments, arising from the malleolar fossa of the fibula and inserting on the posterolateral aspect of the talus and occasionally the os trigonum, with some of its fibers contributing to the formation of the tunnel of the flexor hallucis longus tendon [21].

**Figure 3:**
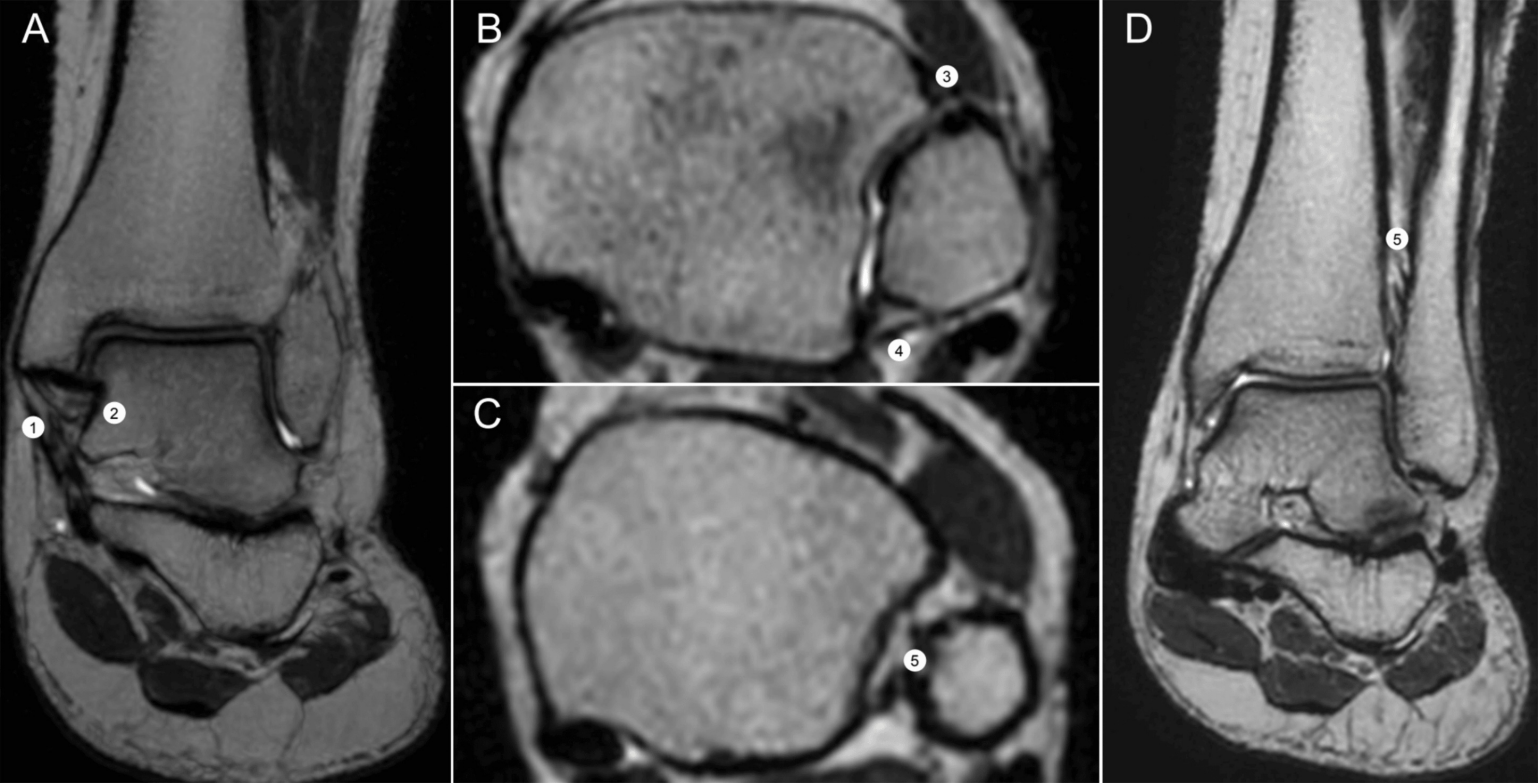
Medial stabilizers and syndesmotic ligaments on CUBE MRI. Three-dimensional T2-weighted reconstructions show (A) the deltoid ligament with superficial fibers (1) and deep fibers (2); (B) anterior (3) and posterior (4) tibiofibular syndesmotic ligaments; (C, D) the interosseous ligament (5). Images belong to the authors; patient consent was obtained.

The medial collateral ligament, or deltoid ligament complex, is significantly stronger than its lateral counterpart and originates from the medial malleolus, inserting into the talus, calcaneus, and navicular (Figure 3).

It is composed of superficial and deep layers. The superficial layer includes the tibionavicular, tibiocalcaneal, tibiospring, and superficial tibiotalar fascicles, while the deep layer consists mainly of the posterior tibiotalar ligament and, in some cases, the anterior tibiotalar ligament [22]. Together, these fascicles resist valgus stress, talar eversion, and external rotation. Injuries of the deltoid ligament are less frequent in isolation because of its strength, but when present, they usually occur in association with fractures of the medial malleolus or syndesmotic disruptions.

The syndesmotic complex includes the anterior and posterior tibiofibular ligaments as well as the interosseous ligament, which represents the distal thickening of the interosseous membrane. The anterior tibiofibular ligament extends from the tibial tubercle to the anterior margin of the lateral malleolus, while the posterior tibiofibular ligament has superficial and deep fascicles that secure the posterior aspect of the mortise [9]. The interosseous ligament, located between the distal tibia and fibula, plays a key role in resisting diastasis and stabilizing axial load transfer across the joint. The tibiofibular recess, which normally measures less than 5 mm, can become fluid-filled and distended on MRI; values greater than 12 mm are strongly associated with syndesmotic injury [23]. A summary of the major ankle ligament complexes, their anatomical attachments, and primary functions is presented in Table 2.

**Table 2:** Major ligament complexes of the ankle: anatomy, function, and imaging considerations. The ankle’s ligamentous support is organized into three main complexes: the lateral collateral ligament (LCL), the medial collateral (deltoid) ligament, and the syndesmotic ligament complex. Each plays a distinct role in mechanical stability, and each has specific imaging features that aid diagnosis. Anterior tibiotalar ligament (deep) is an anatomical variant and may be absent in some individuals [6,24]. Reference numbers are in square brackets. * denotes an anatomical variant; the anterior tibiotalar ligament (deep) may be absent in some individuals.

Complex	Ligament	Origin	Insertion	Primary function(s)	Key imaging considerations
Lateral collateral ligament (LCL) complex	Anterior talofibular ligament (ATFL)	Anterior border of fibular malleolus	Lateral neck of talus	Limits anterior talar translation and plantarflexion; stabilizes the talocrural joint	Best seen on axial/coronal MRI; common injury site; thickness 6-10 mm [5,13]
	Calcaneofibular ligament (CFL)	Anterior border of fibular malleolus	Posterolateral calcaneus	Stabilizes the talocrural and subtalar joints; resists inversion	Visualized in coronal MRI; diameter 6-8 mm, length ≈20 mm [6]
	Posterior talofibular ligament (PTFL)	Fibular malleolar fossa	Posterolateral talus ± os trigonum	Limits posterior talar translation; supports posterior ankle stability	Axial MRI shows relation to the flexor hallucis longus tunnel [6]
Medial collateral (deltoid) ligament complex	Tibionavicular	Medial malleolus	Navicular bone	Resists valgus stress and eversion	Best seen in coronal MRI
	Tibiocalcaneal	Medial malleolus	Sustentaculum tali (calcaneus)	Stabilizes the medial ankle mortise	Coronal MRI evaluation
	Spring (plantar calcaneonavicular)	Sustentaculum tali	Plantar navicular	Supports the medial longitudinal arch	Important in flatfoot deformity
	Tibiospring	Medial malleolus	Blends with the spring ligament	Reinforces arch support	MRI shows blending fibers
	Superficial tibiotalar	Medial malleolus	Medial talar body	Limits eversion/external rotation	Seen in axial/coronal MRI
	Posterior tibiotalar (deep)	Medial malleolus	Medial tubercle of talus	Main restraint to talar external rotation	Axial MRI optimal
	Anterior tibiotalar (deep)*	Medial malleolus	Medial talar body	Resists talar eversion	Anatomical variant*
Syndesmotic ligament complex	Anterior tibiofibular ligament	Anterior tibial tubercle	Anterior fibular malleolus	Resists external rotation and diastasis of the mortise	Axial MRI shows fiber continuity [12]
	Posterior tibiofibular ligament	Posterior fibular malleolus	Posterior tibial tubercle	Maintains posterior mortise stability	Seen in axial MRI
	Interosseous ligament	Medial fibular surface	Lateral tibial surface	Maintains mortise congruence; transmits axial load	MRI: normal tibiofibular recess ≤5 mm; >12 mm suggests injury [7]

Tendinous anatomy

**Figure 4:**
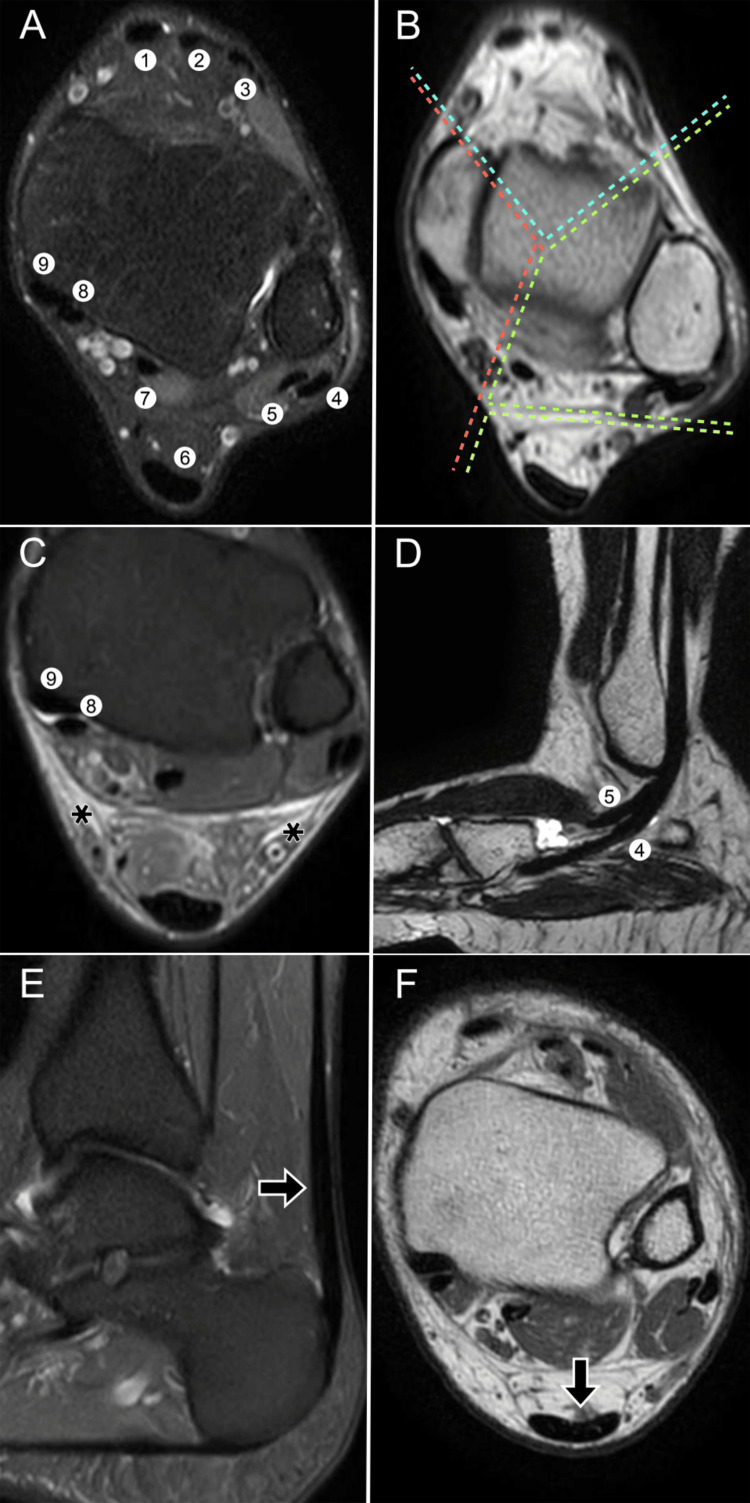
Tendinous compartments of the ankle on MRI. (A) Axial DPFS MRI (dual proton fat-saturated sequence) at the tibio-talar syndesmosis shows (1) tibialis anterior, (2) extensor hallucis longus, (3) extensor digitorum longus, (4) peroneus longus, (5) peroneus brevis, (6) calcaneal (Achilles) tendon, (7) FHL tendon, (8) flexor digitorum longus, and (9) tibialis posterior. (B) The right panel (axial T2 MRI at the talar dome) divides anterior (blue), medial (orange), lateral (green), and posterior (yellow) compartments. (C-D) Axial DPFS MRI shows the posterior tibial (9) and flexor digitorum longus (8) tendons with tenosynovitis (*) and surrounding edema; the right panel shows a multiplanar reconstruction of the peroneus longus (4) and brevis (5). (E-F) Sagittal DPFS MRI depicts the Achilles tendon; axial T2 MRI confirms its normal hypointense signal and semilunar morphology (arrow). Images belong to the authors; patient consent was obtained.

The tendons surrounding the ankle provide dynamic stability, working in synergy with the static stabilizers to preserve joint function during gait, balance, and athletic movements. Structurally, they are composed of collagen, elastin, and reticulin fibers, conferring tensile strength and elasticity. Except for the Achilles tendon, all are enveloped by synovial sheaths that facilitate smooth gliding and are common sites of inflammatory or degenerative change [17]. A compartmental overview of these tendons and their typical imaging appearance is illustrated in Figure 4, which complements the descriptive text and facilitates anatomical localization in daily practice.

In the anterior compartment, the tibialis anterior, extensor hallucis longus, and extensor digitorum longus act as the primary dorsiflexors of the ankle. The tibialis anterior is the strongest and most medial tendon in this group, inserting on the medial cuneiform and the base of the first metatarsal, and plays a crucial role in maintaining the medial longitudinal arch. The extensor hallucis longus inserts on the distal phalanx of the hallux, contributing to both dorsiflexion and hallux extension, whereas the extensor digitorum longus divides into slips to the lateral four toes, providing dorsiflexion and digital extension.

Medially, the tibialis posterior, flexor digitorum longus, and flexor hallucis longus form the principal plantarflexors and invertors of the foot. The tibialis posterior is particularly important for maintaining medial stability and arch support, inserting broadly on the navicular, cuneiforms, cuboid, and bases of the second through fourth metatarsals. The flexor digitorum longus inserts on the distal phalanges of the lateral four toes and crosses the flexor hallucis longus at the “knot of Henry,” a clinically significant site of entrapment and pain syndromes. The flexor hallucis longus inserts on the distal phalanx of the hallux, passes through three fibro-osseous tunnels, and is frequently implicated in posterior ankle impingement syndromes.

The lateral compartment contains the peroneus longus and brevis, both of which evert and plantarflex the foot. The peroneus longus inserts on the plantar surface of the medial cuneiform and first metatarsal, thereby supporting the transverse arch, whereas the peroneus brevis inserts on the base of the fifth metatarsal and is a common site of longitudinal tears.

Posteriorly, the Achilles tendon, formed by the confluence of the gastrocnemius and soleus muscles, inserts on the posterior calcaneal tuberosity. It is the strongest tendon in the human body and the most important plantarflexor of the ankle. Its lack of a synovial sheath and reliance on a paratenon make it prone to degenerative change and rupture, particularly in athletes and older individuals.

Ultrasound is highly effective for evaluating tendinous pathology, offering dynamic assessment and the ability to detect synovial thickening, subluxation, or tears in real time. MRI provides superior contrast resolution and is particularly valuable for assessing deep tendons, detecting tendinopathy, and evaluating associated bone marrow or ligamentous pathology [8,10,24]. A comprehensive summary of the tendinous compartments of the ankle, their insertions, functions, and clinically relevant features is presented in Table 3, which should be read in conjunction with Figure 4 for rapid clinicoradiologic correlation.

**Table 3:** Tendons of the ankle: anatomy, function, and key features. The tendons of the ankle are organized into four compartments based on their anatomical course around the joint. Each tendon’s function is closely related to its compartmental biomechanics and is a key focus in the imaging evaluation of acute and chronic ankle pathologies [8,10].

Compartment	Tendon	Insertion	Primary function(s)	Key anatomical/clinical features
Anterior (extensors)	Tibialis anterior	Medial cuneiform and base of the first metatarsal	Dorsiflexion of the ankle; inversion of the foot	Strongest and most medial tendon in this compartment; supports the medial longitudinal arch; most commonly injured anterior tendon
	Extensor hallucis longus	Dorsal base of the distal phalanx of the hallux	Extension of the distal phalanx of the hallux	Crosses deep to the tibialis anterior; important for hallux dorsiflexion in gait
	Extensor digitorum longus	Four slips to toes 2-5: central slip to base of middle phalanx, lateral slips to distal phalanges	Dorsiflexion of the ankle; extension of toes 2-5	The frequent site of tenosynovitis in runners
Medial (flexors)	Tibialis posterior	Navicular tuberosity, sustentaculum tali, plantar surfaces of cuneiforms, cuboid, bases of second to fourth metatarsals	Plantarflexion; inversion of the foot; medial arch support	Shares tarsal tunnel with flexor digitorum longus; main medial stabilizer of the ankle
	Flexor digitorum longus	Bases of distal phalanges of toes 2-5	Flexion of toes; assists plantarflexion and inversion	Crosses the flexor hallucis longus at the knot of Henry; may be entrapped in the tarsal tunnel
	Flexor hallucis longus	Base of the distal phalanx of the hallux, between the sesamoids	Flexion of hallux; assists plantarflexion	Passes through three fibro-osseous tunnels; the key tendon in posterior ankle impingement
Lateral (evertors)	Peroneus longus	Plantar surface and lateral base of the medial cuneiform and the first metatarsal	Plantarflexion; eversion; transverse arch support	Oval morphology; traverses peroneal groove; visualized on axial MRI and ultrasound
	Peroneus brevis	Base of the fifth metatarsal	Eversion; assists plantarflexion	Flattened morphology; frequent site of longitudinal split tears
Posterior (plantarflexor)	Achilles (calcaneal) tendon	Posterior calcaneal tuberosity	Powerful plantarflexion; propulsion in gait	The thickest tendon in the human body; no synovial sheath, surrounded by paratenon; prone to degenerative tendinopathy and rupture

Traumatic pathology

Modality selection is guided by clinical suspicion and mechanism of injury; a practical comparison of strengths and limitations is provided in Table 1 to streamline imaging decisions in acute and subacute settings [3,5,15,16].

**Figure 5:**
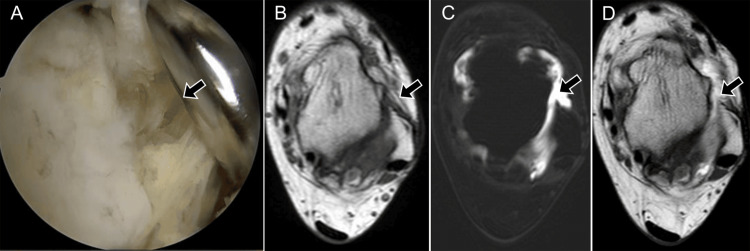
Chronic anterolateral ankle instability - arthroscopy and MRI. A 45-year-old female: (A) arthroscopic view via an ATFL approach shows absence of ATFL fibers (arrow); (B) axial T2 MRI without fat suppression shows irregular, thickened ATFL with mild hyperintensity (arrow); (C) post-contrast axial T1 fat-saturated MRI shows contrast leakage into the anterolateral recess (arrow); (D) axial T1 spin-echo MRI confirms ligament discontinuity (arrow). ATFL: anterior talofibular ligament Images belong to the authors; patient consent was obtained.

Ankle sprains are the most common traumatic injuries during recreational and sports activities, accounting for 16-40% of all sports-related injuries [3]. The typical mechanism involves inversion combined with plantarflexion and adduction. These injuries are associated with high recurrence, and chronic ankle instability develops in 10-40% of cases, representing a major cause of functional limitation in young and athletic populations [25]. Examples of lateral ligament injury are demonstrated in Figure 5.

**Figure 6:**
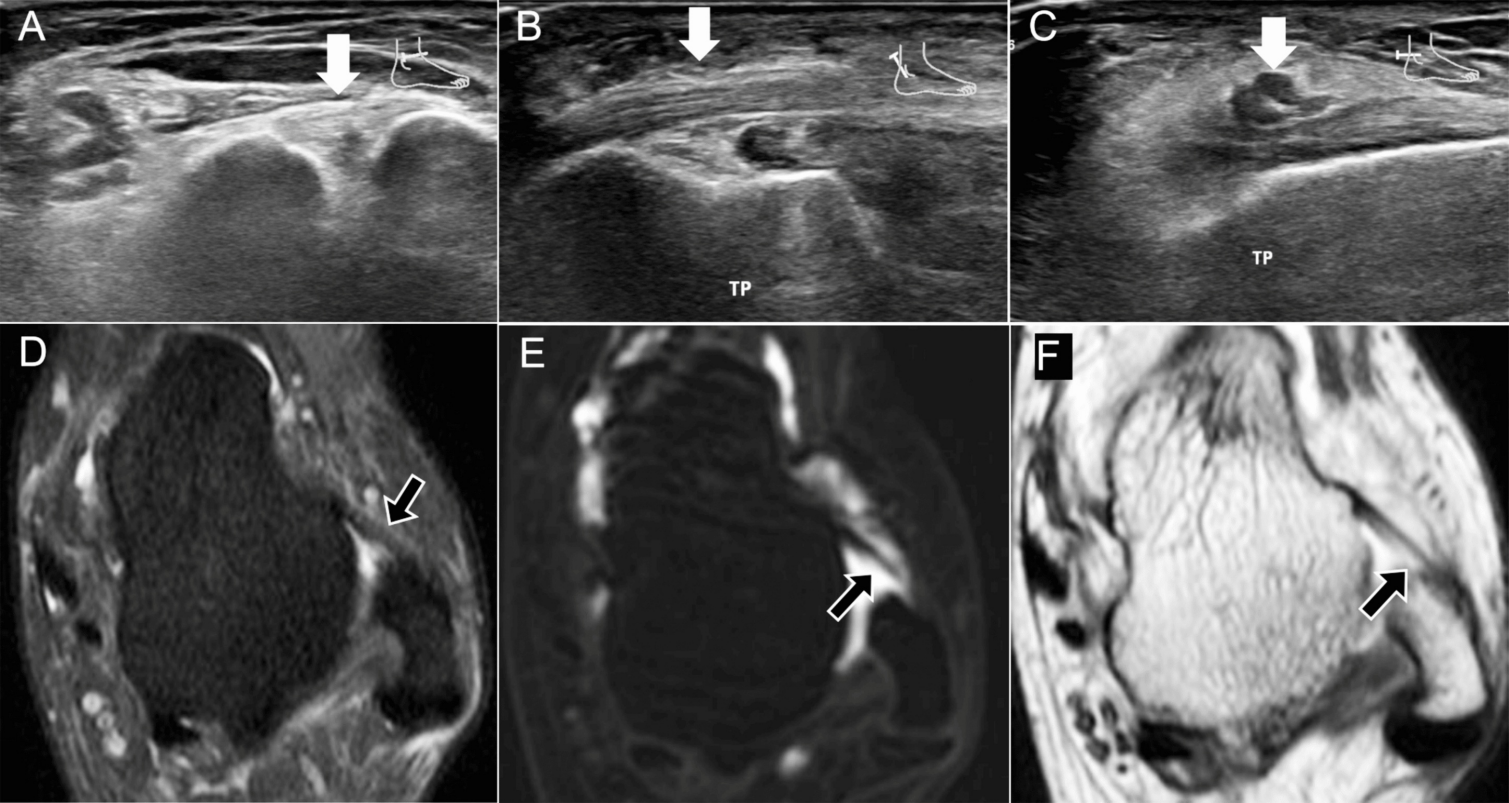
Ultrasound and MRI of ATFL tear with intact peroneal tendons. A 32-year-old female with chronic instability: (A) transverse ultrasound shows a full-thickness (Grade III) ATFL tear (arrow); (B, C) longitudinal and transverse ultrasound at the retromalleolar level show intact peroneal tendons (arrows); (D) pre-contrast DPFS MRI shows a thickened, hyperintense ATFL (arrow); (E) post-contrast T1 SPGR MR arthrography (spoiled gradient-echo) shows loss of fibular insertion continuity (arrow); (F) post-contrast T1 spin-echo MRI confirms an ATFL tear with a few residual fibers (arrow). AFTL: anterior talofibular ligament; DPFS: dual proton fat-saturated sequence; SPGR: spoiled gradient-echo Images belong to the authors; patient consent was obtained.

**Figure 7:**
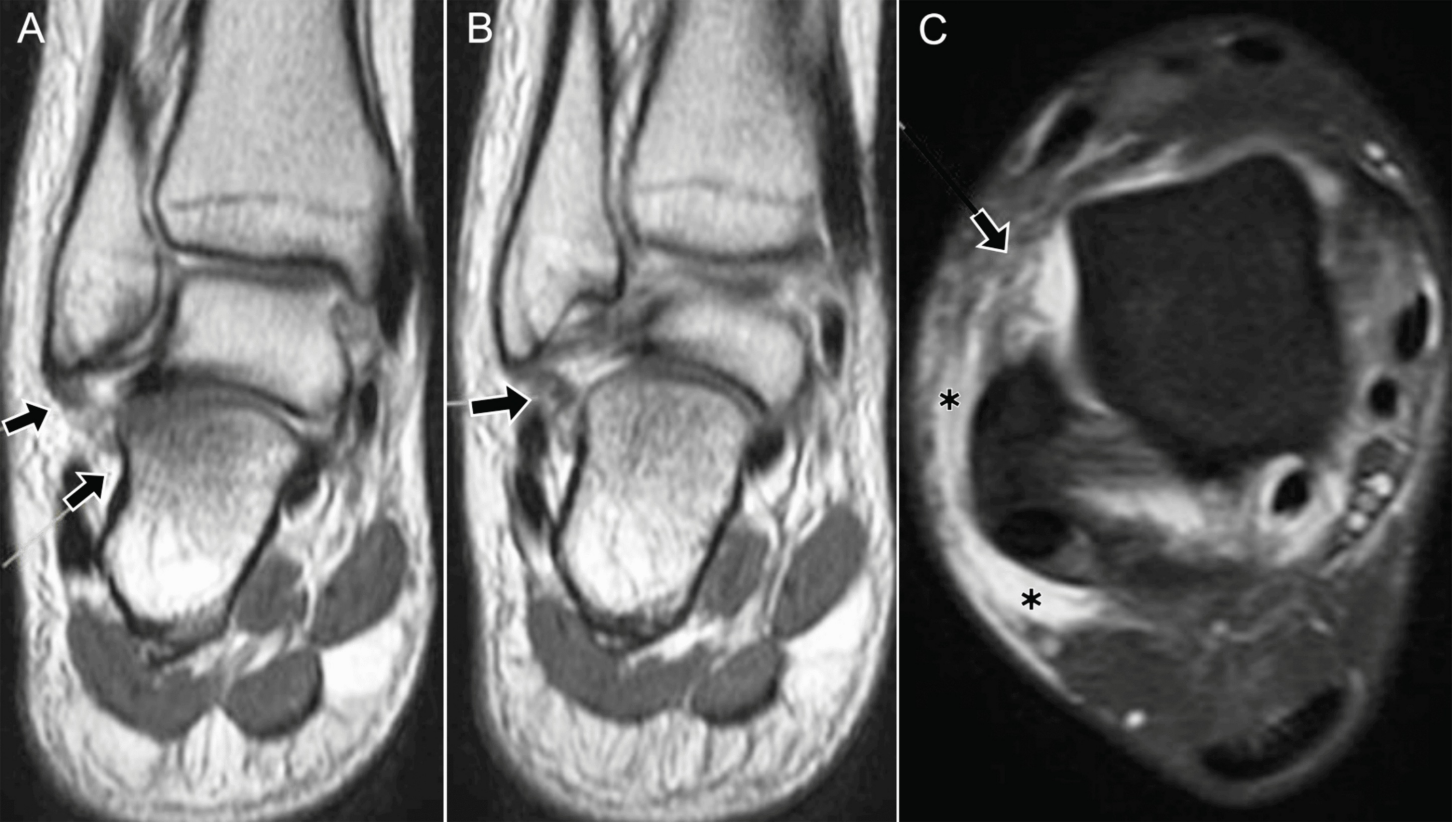
Calcaneofibular and ATFL tears after trauma. A 22-year-old female: (A, B) coronal DP MRI (double-echo proton) reveals full-thickness CFL tears (arrows); (C) axial DPFS MRI demonstrates a complete ATFL tear (arrow) and lateral soft-tissue injury (*). CFL: calcaneofibular ligament; DPFS: dual proton fat-saturated sequence; ATFL: anterior talofibular ligament Images belong to the authors; patient consent was obtained.

**Figure 8:**
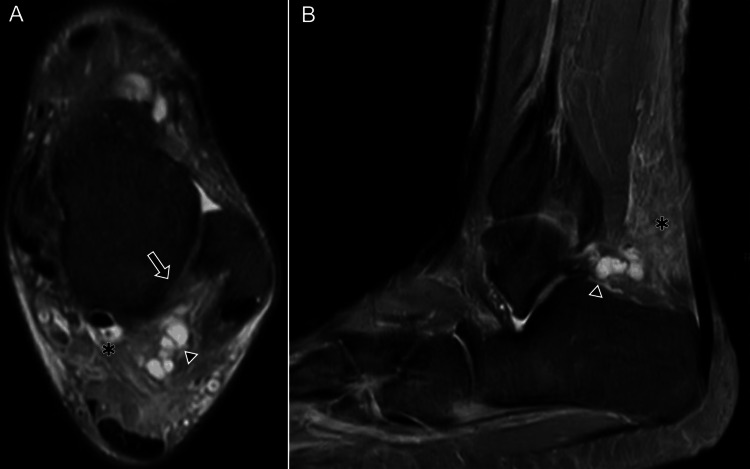
Posterior talofibular ligament (PTFL) injury with organized ganglion. A 43-year-old male: (A) axial DPFS MRI shows a thickened PTFL (arrow), a multiseptated ganglion (arrowhead), and fluid within the FHL tendon sheath (*); (B) sagittal DPFS MRI shows the organized ganglion (arrowhead) adjacent to the PTFL and heterogeneous edema of Kager’s fat pad (*). DPFS: dual proton fat-saturated sequence; FHL: flexor hallucis longus Images belong to the authors; patient consent was obtained.

The LCL complex is the structure most frequently injured, involved in up to 85% of ankle sprains [26]. Within this complex, the ATFL is the most commonly torn (Figure 6), followed by the calcaneofibular ligament (CFL) (Figure 7), while the PTFL is least frequently affected (Figure 8).

**Figure 9:**
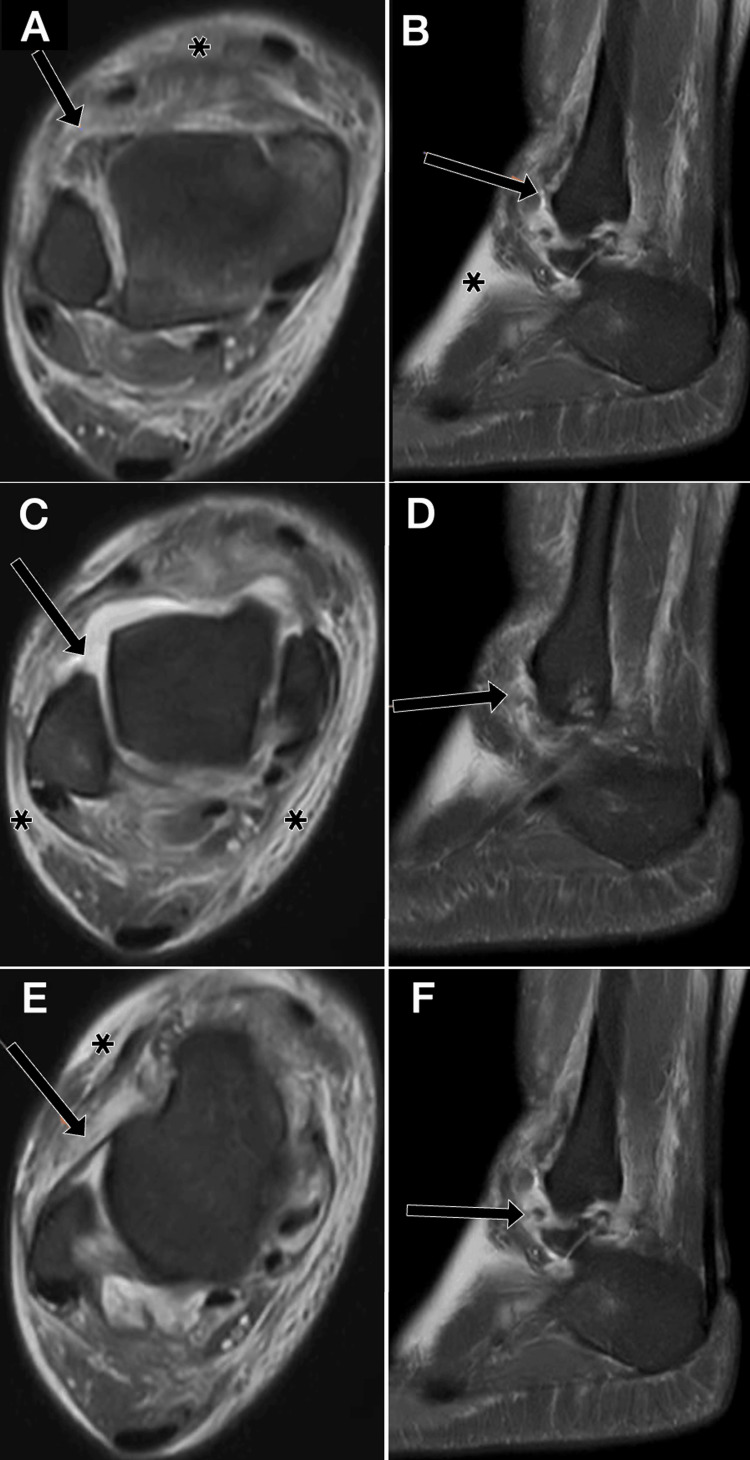
Syndesmotic and anterior talofibular ligament injuries following inversion trauma in a 37-year-old male (DPFS MRI). (A) Axial image at the level of the distal tibiofibular syndesmosis showing a tear of the anterior tibiofibular ligament (black arrow). (B) Sagittal image through the fibula demonstrating the anterior tibiofibular ligament (black arrow) with adjacent soft-tissue edema (*). (C) Axial image at the proximal talus showing a complete tear of the superior fibers of the anterior talofibular ligament (ATFL) (black arrow) with surrounding soft-tissue edema (*). (D) Sagittal image at the distal fibula showing a tear of the ATFL (black arrow). (E) Axial image demonstrating thinned inferior fibers of the ATFL (black arrow) with periligamentous edema (*). (F) Sagittal image at the distal fibula showing residual ATFL fibers (black arrow) with surrounding edema (*). ATFL: anterior talofibular ligament; DPFS: dual proton fat-saturated sequence Images belong to the authors; patient consent was obtained.

**Figure 10:**
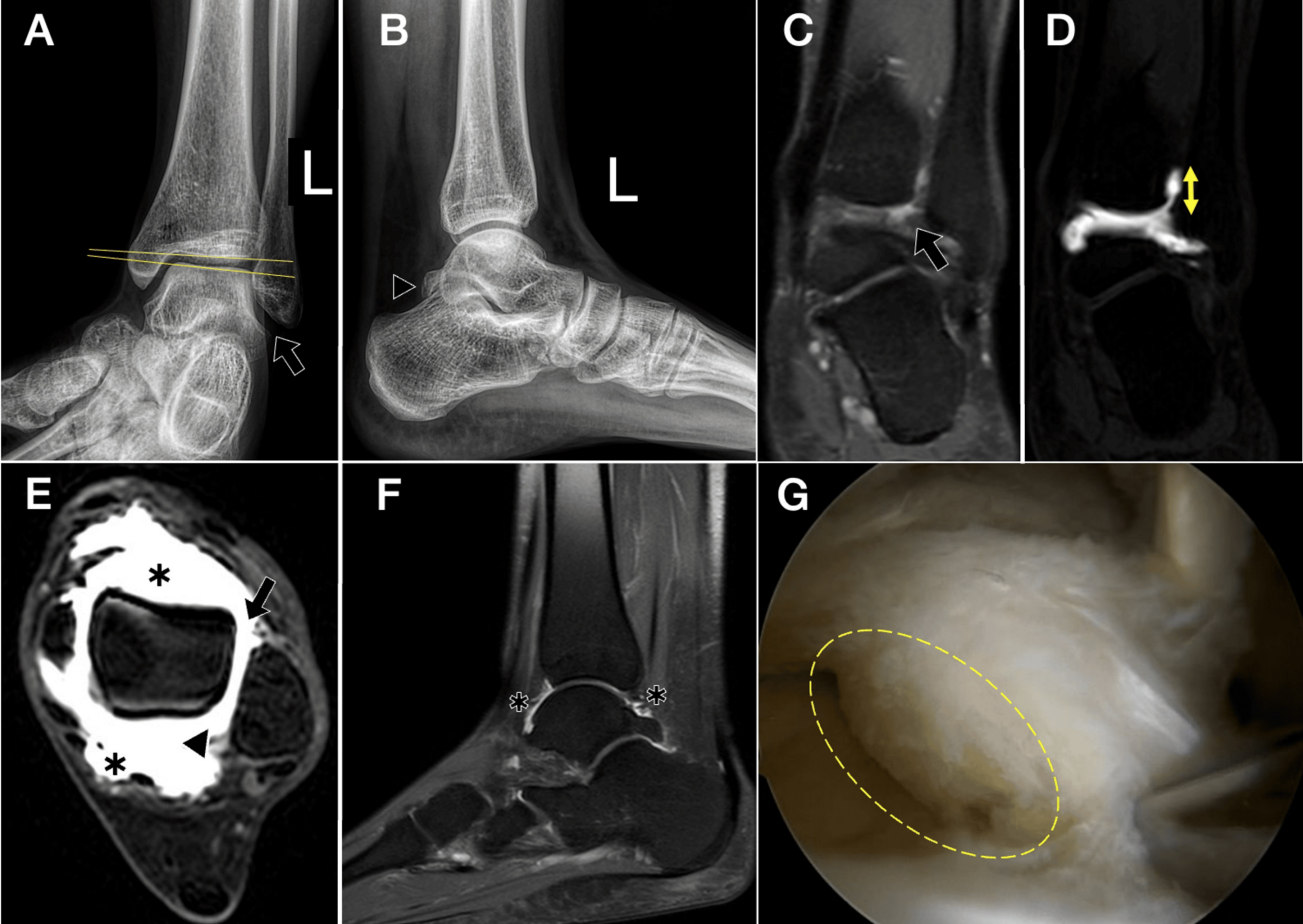
Imaging findings in anterolateral ankle instability in a 36-year-old female. (A) Anteroposterior (AP) radiograph showing loss of tibio-talar parallelism with widening of the lateral clear space (arrow). (B) Lateral radiograph demonstrating normal osseous alignment in this projection and a prominent Stieda process (black arrowhead). (C) Coronal STIR MRI at the level of the syndesmosis showing interposed edema within the distal tibiofibular syndesmosis (black arrow). (D) Coronal post-contrast T1 fat-suppressed MRI after intra-articular contrast (direct MR arthrography) demonstrating contrast ascending into the distal tibiofibular syndesmosis >7 mm above the joint line (double-headed yellow arrow). (E) Axial T1 spin-echo fat-suppressed image after intra-articular contrast: anterior and posterior talar recesses are distended with contrast (*). The anterior arrow indicates a full-thickness ATFL tear (absence of fibers); the arrowhead marks contrast at the level of the PTFL. (F) Sagittal pre-contrast STIR image through the tibiotalar joint showing enlarged anterior and posterior recesses (*) associated with a prominent Stieda process. (G) Arthroscopic image (posterior approach) in the same patient demonstrating the Stieda process with fibers of the PTFL and entrapment of the FHL tendon (yellow dashed ellipse). ATFL: anterior talofibular ligament; FHL: flexor hallucis longus; MR: magnetic resonance; PTFL: posterior talofibular ligament; STIR: short tau inversion recovery Images belong to the authors; patient consent was obtained.

**Figure 11:**
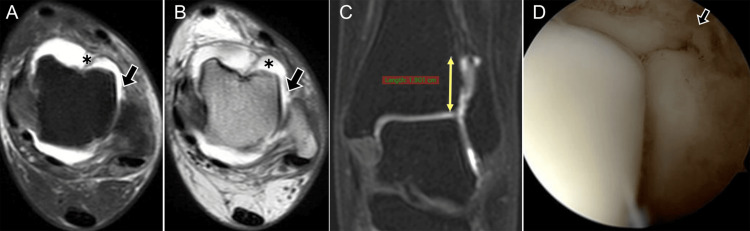
Combined syndesmotic and ATFL injury. (A) Axial STIR MRI (short tau inversion recovery) and (B) post-contrast T1 spin-echo MRI show a full-thickness ATFL tear (arrow) with contrast leakage (*); (C) coronal T1 fat-suppressed MRI shows contrast extending 1.9 cm above the syndesmosis (yellow line); (D) arthroscopy confirms ATFL rupture (arrow). ATFL: anterior talofibular ligament Images belong to the authors; patient consent was obtained.

**Figure 12:**
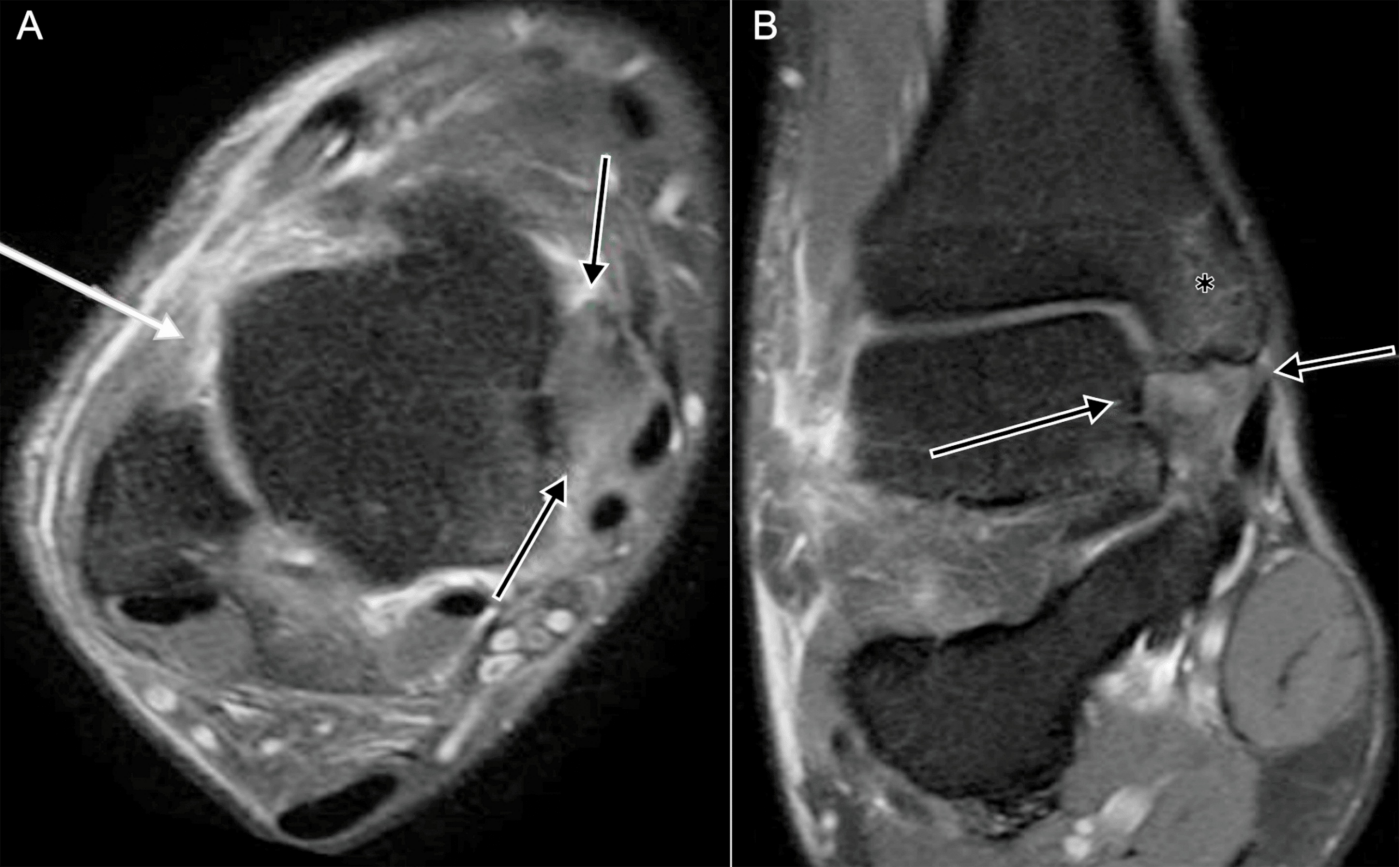
Anterolateral ligament and deltoid complex injury on MRI. A 16-year-old male: (A) axial DPFS MRI shows discontinuity of the anterolateral ligament (white arrow) and a partial tear of the deep deltoid fibers (black arrows); (B) coronal DPFS MRI demonstrates a deep deltoid fiber tear (black arrows) and tibial marrow edema (*). DPFS: dual proton fat-saturated sequence Images belong to the authors; patient consent was obtained.

**Figure 13:**
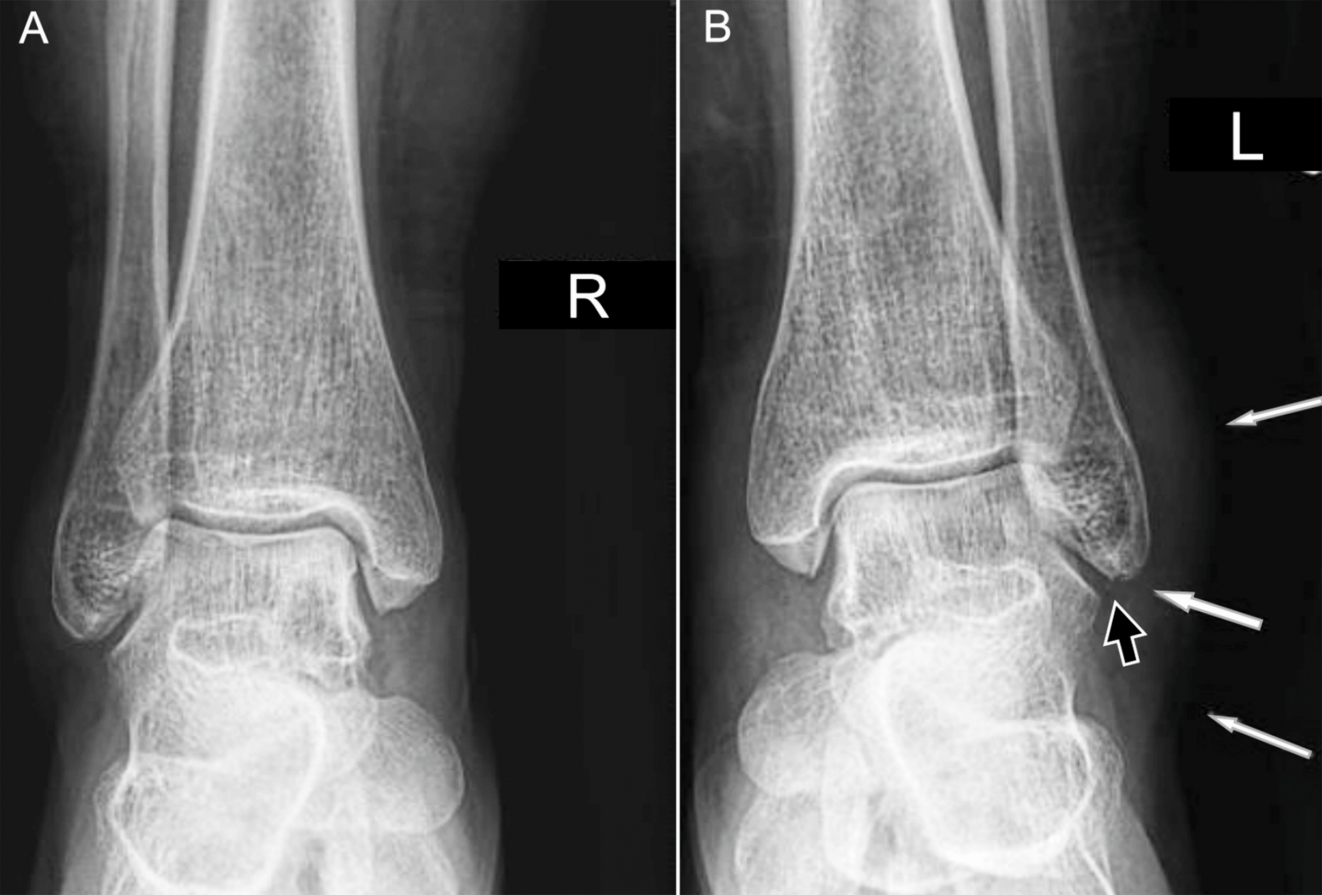
Comparative ankle radiographs after trauma. A 60-year-old female: (A) anteroposterior radiograph of the right ankle shows normal alignment and soft tissues; (B) anteroposterior radiograph of the left ankle shows cortical discontinuity at the fibular apex (black arrow) with lateral soft-tissue swelling (white arrows). Images belong to the authors; patient consent was obtained.

Injuries to the LCL are often associated with damage to other compartments, including syndesmotic disruption (Figures 9-11), osteochondral lesions, medial collateral ligament involvement (Figure 12), sinus tarsi ligament tears, peroneal tendon pathology, or fractures, which are best demonstrated on plain radiographs (Figure 13) [16].

**Figure 14:**
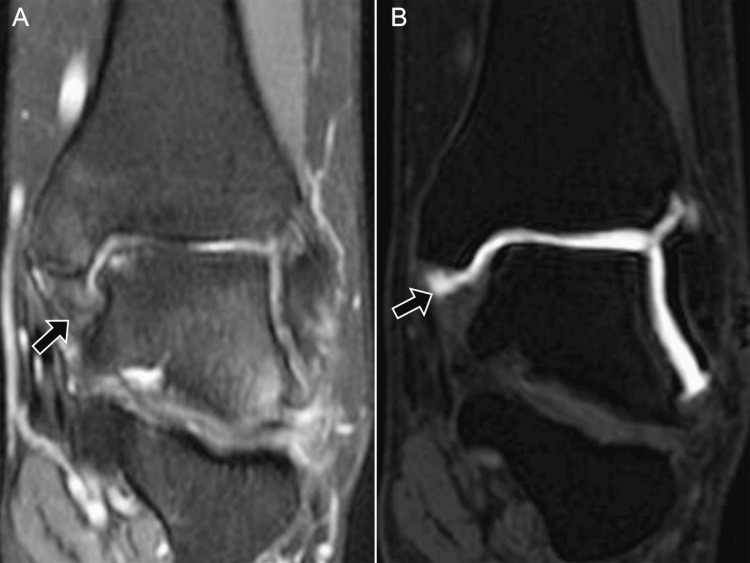
MR arthrography revealing deltoid ligament tear. A 40-year-old male: (A) coronal non-contrast T2 fat-saturated MRI shows a thickened, hyperintense deltoid ligament (arrow); (B) coronal post-contrast T1 fat-saturated MR arthrography reveals a linear hyperintensity indicating a full-thickness tear of the deep deltoid fibers with contrast extension (arrow). Images belong to the authors; patient consent was obtained.

**Figure 15:**
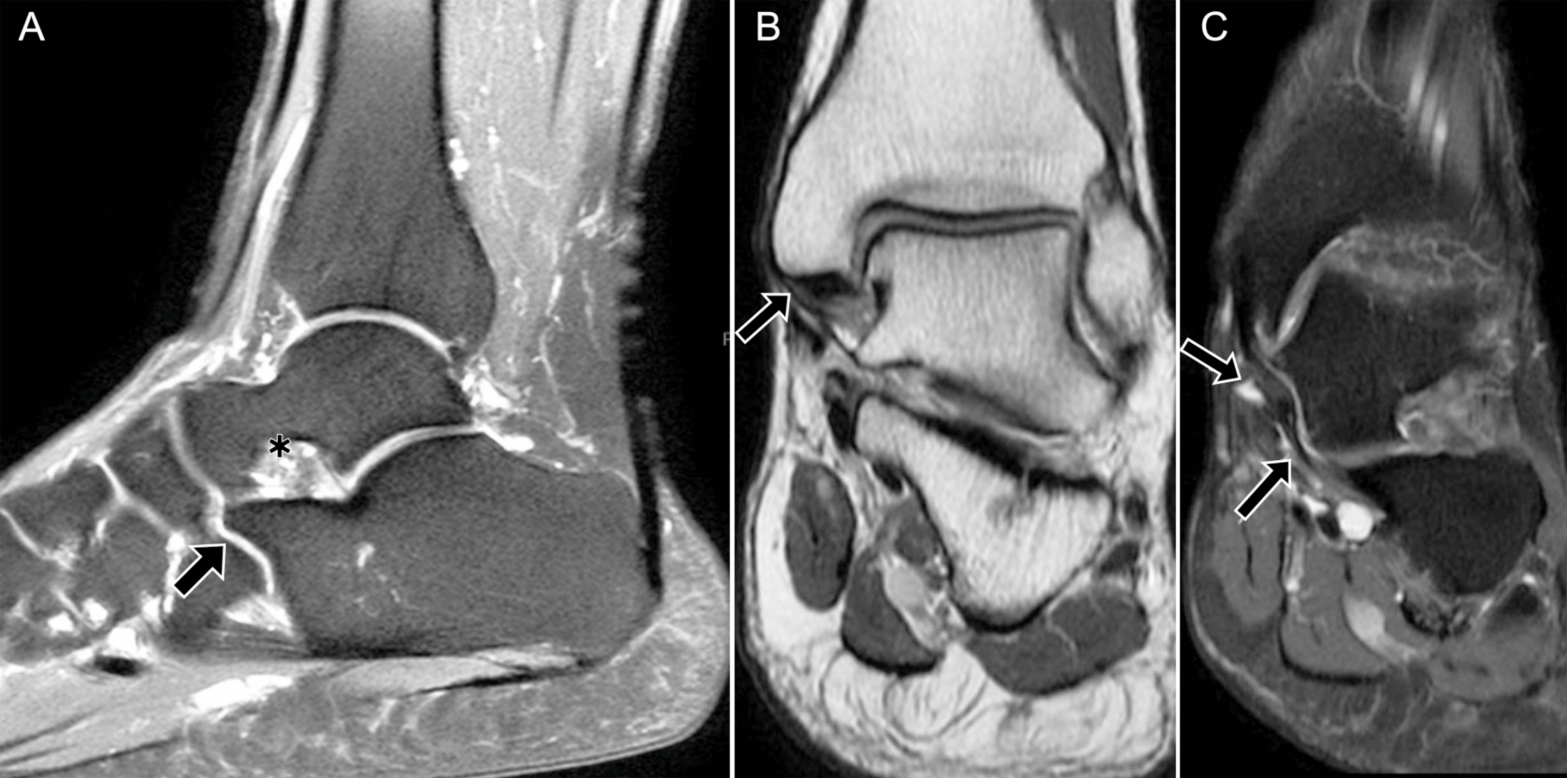
Deltoid and tibiospring ligament injuries. A 43-year-old female: (A) sagittal T2 fat-saturated MRI shows a heterogeneous sinus tarsi (*) and fluid in the calcaneocuboid joint (black arrow); (B) coronal DP MRI (double-echo proton) shows the tibiospring ligament origin (black arrow); (C) coronal DPFS MRI shows a partial superficial tibiospring tear with increased signal and laxity (arrows). DPFS: dual proton fat-saturated sequence Images belong to the authors; patient consent was obtained.

Isolated injury of the medial collateral ligament (deltoid complex) is rare due to its greater tensile strength compared with the lateral ligaments. Early diagnosis is crucial, as the stability of the talus depends largely on this ligamentous complex, with the deep fascicle being most important [27]. Deltoid ligament injuries are usually secondary to pronation or eversion mechanisms. Most cases involve avulsion of the tibial insertion, producing medial talar displacement with widening of the medial clear space greater than 4 mm. In addition, deltoid injuries are frequently associated with concomitant LCL tears in approximately 75% of cases, syndesmotic injury in 10%, and fractures, such as supination-external rotation or Weber B type, in 20-50% [18]. These injuries are illustrated in Figures 14-15.

**Figure 16:**
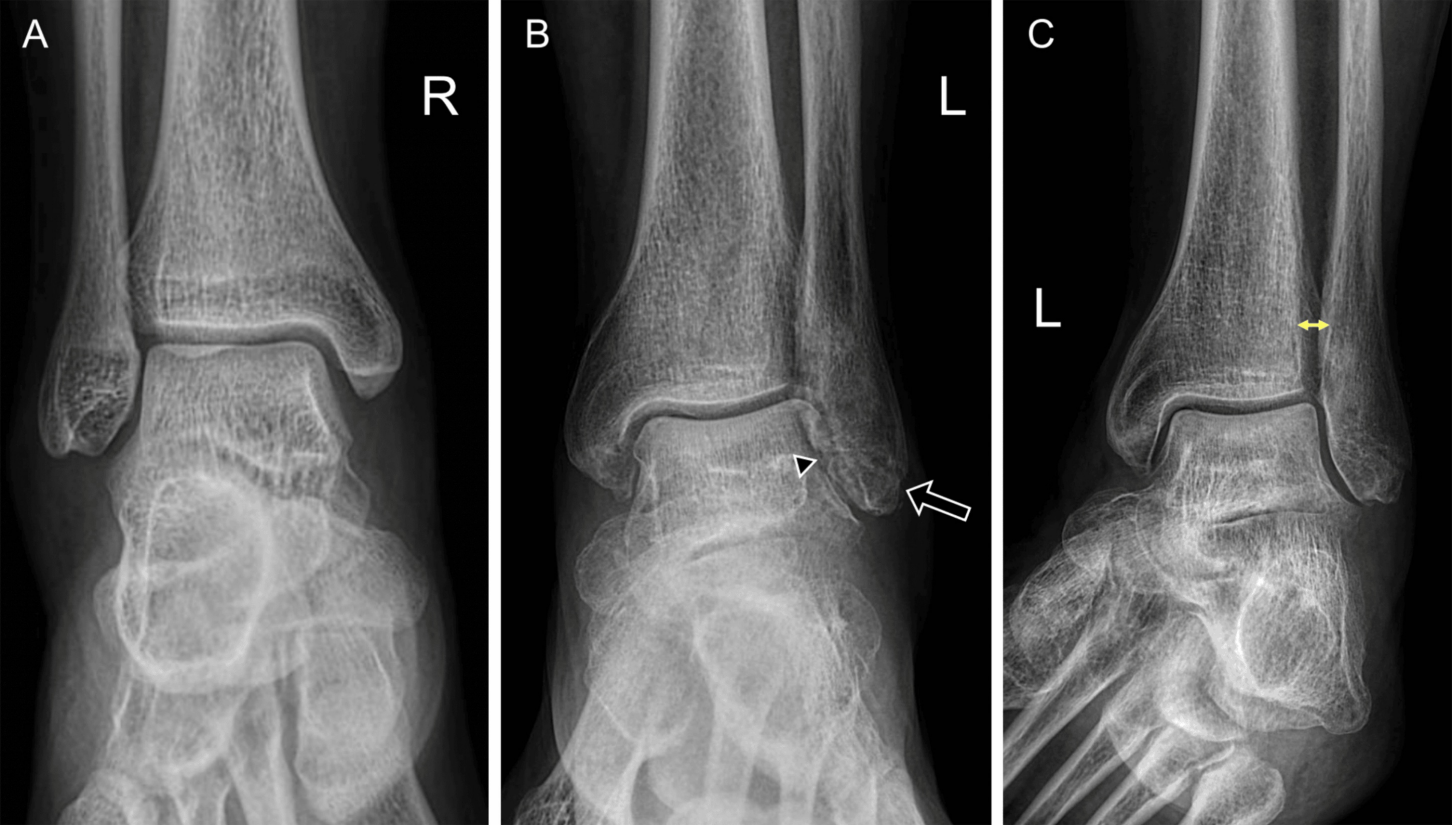
Radiographic appearance of syndesmotic widening. (A) Anteroposterior radiograph of a 19-year-old female demonstrates normal syndesmotic width; (B) anteroposterior radiograph of a 67-year-old female shows a distal fibula fracture (arrow) and decreased lateral joint space (arrowhead); (C) mortise projection of the same ankle shows a widened syndesmotic space (double arrow). Images belong to the authors; patient consent was obtained.

High ankle sprains, also referred to as distal tibiofibular syndesmotic injuries, account for approximately 10% of ankle sprains [28]. They are often accompanied by ATFL injury, bone contusions, or fractures. The most widely accepted mechanism is external rotation of the foot combined with internal rotation of the leg, usually following high-energy trauma. Conventional radiography remains the first-line imaging modality, providing indirect signs of syndesmotic instability. Diagnostic markers include a tibiofibular clear space greater than 5 mm in the anteroposterior view, with a sensitivity of 82% and a specificity of 75%. A tibiofibular overlap of less than 6 mm on the anteroposterior view and less than 2.8 mm on the mortise view (Figure 16) has a sensitivity of 36% and specificity of 87% [15].

Osteochondral lesions of the talar dome occur in up to 7% of patients following ankle sprain and more often affect the medial dome [29]. These injuries are typically caused by compressive or rotational mechanisms. Radiography does not allow reliable detection of cartilage defects, although indirect signs such as subchondral cysts or loose bodies may raise suspicion. More than 50% of osteochondral lesions cannot be diagnosed by plain radiographs alone [30]. MRI is therefore the modality of choice, using high-resolution sequences such as three-dimensional (3D), double proton density (DP), T2-weighted, sampling perfection with application-optimized contrasts using different flip angle evolution with fat suppression (SPACE FS), and T2, which enable optimal assessment of cartilage and subchondral bone.

The most widely used classification for osteochondral lesions of the talus (OLT) is the Berndt and Harty system, modified by Loomer [31]. The Mintz classification [19] correlates MRI findings with arthroscopic appearance, enhancing clinical applicability. According to Berndt and Harty, lesions are graded as stage I (subchondral compression injury), stage IIa (subchondral cyst), stage IIb (incomplete fragment separation), stage III (complete separation without displacement), and stage IV (displaced fragment). Osteochondral lesions of traumatic etiology are more commonly located on the lateral talar dome, while degenerative lesions tend to involve the medial dome [29]. Correct localization and extension of the lesion are crucial for treatment planning.

**Figure 17:**
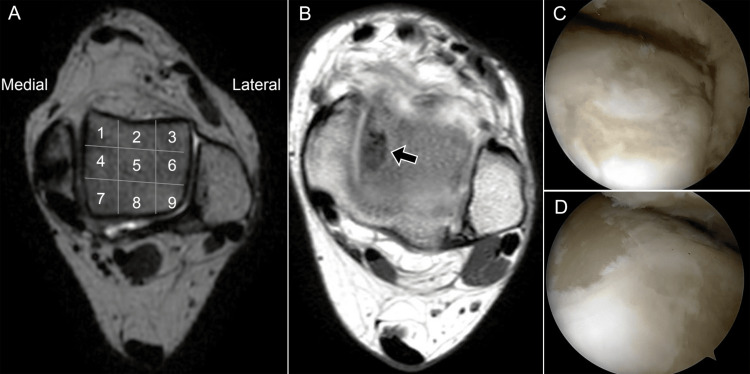
Localization of osteochondral talar lesions. (A) Axial T2 MRI shows the Raikin et al. grid for lesion localization; (B) axial T2 MRI shows chondral denudation in zone 4 (arrow); (C, D) arthroscopy confirms the lesion in the corresponding medial talar dome region. Images belong to the authors; patient consent was obtained.

Raikin and colleagues proposed a nine-zone grid, arranged in a 3 × 3 configuration, for precise mapping of OLTs in MRI (Figure 17). This classification provides a reproducible method to aid surgical planning, intraoperative visualization, and postoperative follow-up [29].

Degenerative pathology

Tibiotalar and Subtalar Osteoarthritis

**Figure 18:**
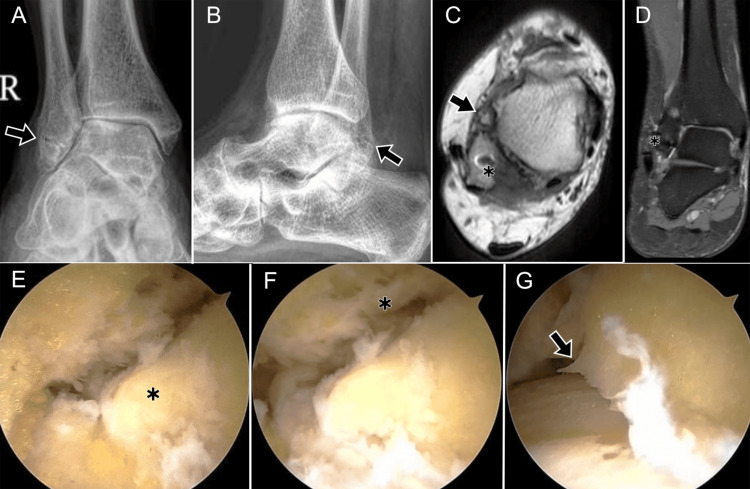
Chronic instability and tibio-talar osteoarthritis. A 66-year-old male: (A, B) anteroposterior and lateral weight-bearing radiographs show sclerosis, surface irregularity, and marginal osteophytes indicating osteoarthritis (arrows); (C, D) axial DP MRI (double-echo proton) and coronal STIR MRI reveal distal fibula surgical changes (asterisk), osteophytes, an ossicle in the ATFL recess, and a full-thickness ATFL tear (arrow); (E-G) arthroscopy shows synovial thickening (asterisk), tibiofibular instability (variable amplitude), irregular cartilage, and marginal osteophytes (arrow). STIR: short tau inversion recovery; ATFL: anterior talofibular ligament Images belong to the authors; patient consent was obtained.

Osteoarthritis of the ankle (Figure 18) is a chronic, progressive, and irreversible degradation of the articular surface that is often accompanied by joint inflammation. Primary osteoarthritis of the ankle is rare, accounting for less than 1% of cases, while secondary osteoarthritis is far more common and is usually the result of trauma or chronic ankle instability. Patients frequently present with a history of fracture, osteochondral lesions, or inflammatory arthropathies. The latency period between the inciting pathology and the development of osteoarthritis may exceed 20 years in up to 40% of patients [32].

Initial evaluation relies on conventional radiographs obtained in anteroposterior, lateral, and mortise weight-bearing projections. These studies reveal characteristic features such as osteophyte formation, joint space narrowing, subchondral sclerosis, and subchondral cysts. Additional findings may include avulsion fragments, osteonecrosis, or insufficiency fractures. The Kellgren-Lawrence grading system and the Osteoarthritis Research Society International (OARSI) atlas are commonly used for radiographic characterization [33]. 

MRI is considered the modality of choice when a detailed evaluation of the ankle joint is required. MRI can demonstrate joint effusion, synovitis, and trabecular bone marrow edema. Advanced quantitative techniques such as T2 mapping and T1ρ mapping allow indirect assessment of proteoglycan content within the cartilage matrix, providing a non-invasive means of evaluating early degenerative change [14].

Chronic Achilles Tendinopathy

The normal Achilles tendon measures approximately 4-7 mm in thickness, with a concave anterior margin on axial imaging. Its length from the calcaneal insertion is about 5-6 cm. A defining characteristic of this tendon is its relative hypovascularity, particularly in the region 2-6 cm proximal to its insertion, which predisposes it to degenerative injury [23]. Chronic tendinopathy is multifactorial, influenced by both intrinsic and extrinsic factors such as age, overuse, metabolic disease, and biomechanical abnormalities [34].

**Figure 19:**
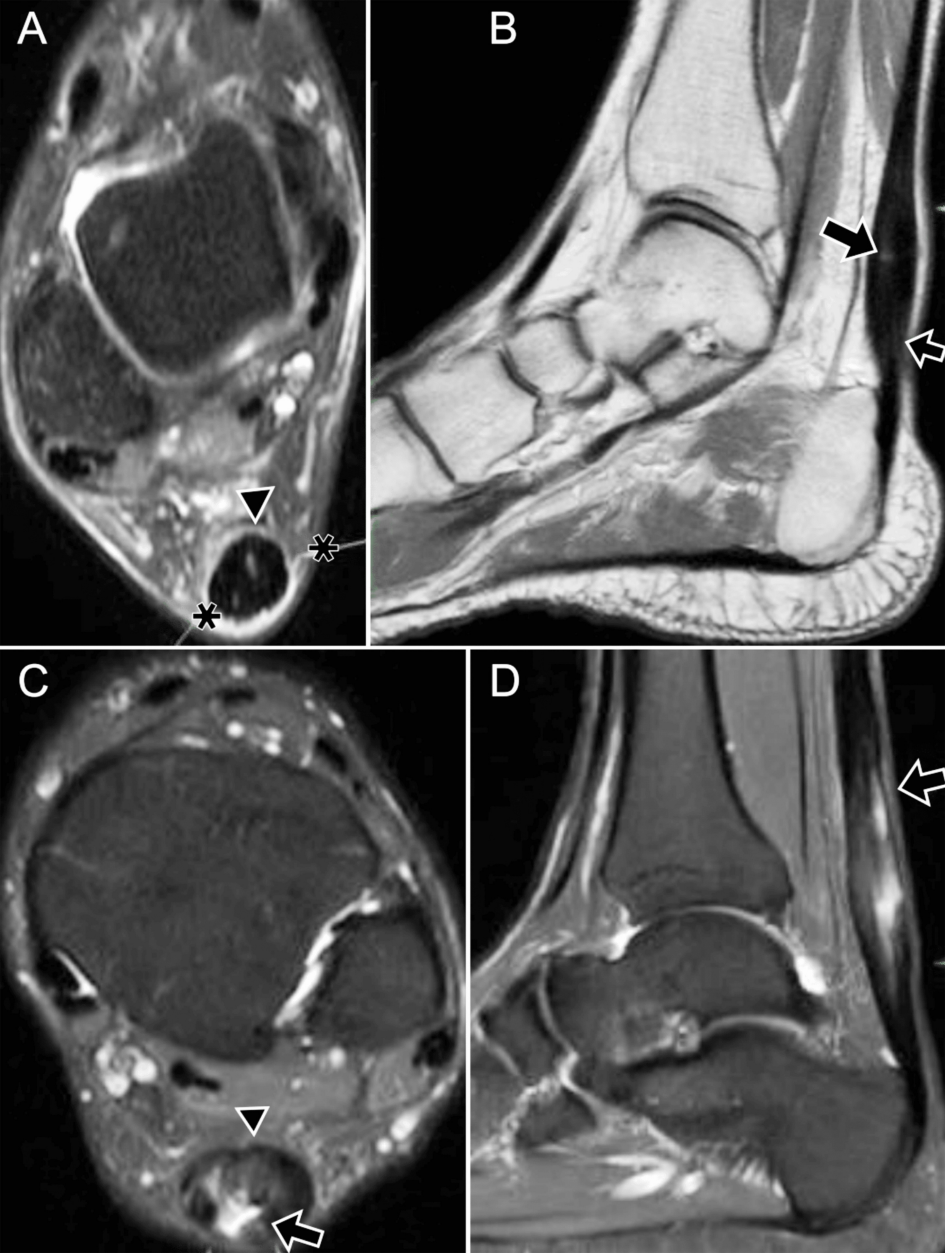
MRI of Achilles tendon injuries. (A) Axial T2 fat-saturated MRI of a 40-year-old female shows heterogeneous intratendinous hyperintensity (arrowhead) with peritendinous edema (asterisks); (B) sagittal T2 MRI of the same patient shows fusiform thickening of the distal Achilles tendon (black arrows); (C) axial DPFS MRI of a 64-year-old female shows loss of anterior concavity (arrowhead) and heterogeneous intrasubstance signal (arrow); (D) axial DPFS MRI of the same patient shows fusiform thickening with intrasubstance hyperintensity (arrow). DPFS: dual proton fat-saturated sequence Images belong to the authors; patient consent was obtained.

MRI is the most sensitive modality for detecting chronic degenerative changes of the Achilles tendon. Insertional tendinopathy manifests as increased signal intensity on fat-suppressed sequences and thickening at the calcaneal attachment. Non-insertional tendinopathy, in contrast, typically involves fusiform thickening of the proximal to mid-tendon with heterogeneous T1 and T2 signal. These changes are demonstrated in Figure 19 [35].

Limitations

As a narrative review, our approach enhances clinical readability but may be less reproducible than systematic methodologies; nevertheless, we mitigated this by reporting our timeframe, databases, last search date, and eligibility filters and by balancing seminal references with recent advances [3,4,6-10,14,15,19-22,29-31].

## Conclusions

A precise understanding of ankle anatomy - osseous architecture, ligamentous complexes, and tendon compartments - is essential to interpret mechanisms of injury and the broad spectrum of traumatic and degenerative disorders. The joint’s compact anatomy and high functional demand explain both its versatility and its susceptibility to sprains, syndesmotic disruption, osteochondral injury, tendinopathy, and post-traumatic osteoarthritis. Modern imaging has transformed evaluation: radiographs remain indispensable in the acute setting, while ultrasound, CT, and MRI progressively enhance soft-tissue and osteochondral assessment. Emerging techniques, including WBCT and high-resolution isotropic or quantitative MRI, detect early or complex abnormalities that may be occult on conventional studies.

Clinical value comes from merging these imaging capabilities with anatomical and biomechanical insight. Accurate diagnosis depends on recognizing injury patterns, correlating symptoms with structures at risk, and appreciating the consequences of missed or delayed findings. By coupling detailed anatomy with practical imaging correlations and arthroscopic confirmation where available, this review supports a multidisciplinary approach that refines diagnostic precision, informs treatment planning, and ultimately improves functional outcomes for patients with ankle disorders.

## References

[1] Manganaro D, Alsayouri K (2023). Anatomy, bony pelvis and lower limb: ankle joint. StatPearls [Internet].

[2] Tanino T, Ogiso T, Iwaki M, Yamaguchi T, Kakehi K (1999). Release characteristics of endogenous constituents by exposure of small intestine to modified beta-cyclodextrins. Biol Pharm Bull.

[3] Jungmann PM, Lange T, Wenning M, Baumann FA, Bamberg F, Jung M (2023). Ankle sprains in athletes: current epidemiological, clinical and imaging trends. Open Access J Sports Med.

[4] Bajaj S, Chhabra A, Taneja AK (2024). 3D isotropic MRI of ankle: review of literature with comparison to 2D MRI. Skeletal Radiol.

[5] Beltran LS, Zuluaga N, Verbitskiy A, Bencardino JT (2023). Imaging of acute ankle and foot sprains. Radiol Clin North Am.

[6] Golanó P, Vega J, de Leeuw PA, Malagelada F, Manzanares MC, Götzens V, van Dijk CN (2010). Anatomy of the ankle ligaments: a pictorial essay. Knee Surg Sports Traumatol Arthrosc.

[7] Perrich KD, Goodwin DW, Hecht PJ, Cheung Y (2009). Ankle ligaments on MRI: appearance of normal and injured ligaments. AJR Am J Roentgenol.

[8] Choplin RH, Buckwalter KA, Rydberg J, Farber JM (2004). CT with 3D rendering of the tendons of the foot and ankle: technique, normal anatomy, and disease. Radiographics.

[9] Bartonícek J (2003). Anatomy of the tibiofibular syndesmosis and its clinical relevance. Surg Radiol Anat.

[10] Cheung Y, Rosenberg ZS, Magee T, Chinitz L (1992). Normal anatomy and pathologic conditions of ankle tendons: current imaging techniques. Radiographics.

[11] Lau BC, Allahabadi S, Palanca A, Oji DE (2022). Understanding radiographic measurements used in foot and ankle surgery. J Am Acad Orthop Surg.

[12] Brockett CL, Chapman GJ (2016). Biomechanics of the ankle. Orthop Trauma.

[13] Tol JL, van Dijk CN (2004). Etiology of the anterior ankle impingement syndrome: a descriptive anatomical study. Foot Ankle Int.

[14] Wu W, Kang Z, Mu D, Zhao H, Yang F (2023). T2 mapping for quantitative assessment of ankle cartilage of weightlifters. Sci Rep.

[15] Pogliacomi F, De Filippo M, Casalini D (2021). Acute syndesmotic injuries in ankle fractures: from diagnosis to treatment and current concepts. World J Orthop.

[16] Staats K, Sabeti-Aschraf M, Apprich S (2018). Preoperative MRI is helpful but not sufficient to detect associated lesions in patients with chronic ankle instability. Knee Surg Sports Traumatol Arthrosc.

[17] De Maeseneer M, Marcelis S, Jager T, Shahabpour M, Van Roy P, Weaver J, Jacobson JA (2009). Sonography of the normal ankle: a target approach using skeletal reference points. AJR Am J Roentgenol.

[18] Mengiardi B, Pinto C, Zanetti M (2016). Medial collateral ligament complex of the ankle: MR imaging anatomy and findings in medial instability. Semin Musculoskelet Radiol.

[19] Mintz DN, Tashjian GS, Connell DA, Deland JT, O'Malley M, Potter HG (2003). Osteochondral lesions of the talus: a new magnetic resonance grading system with arthroscopic correlation. Arthroscopy.

[20] van den Bekerom MP, Oostra RJ, Golanó P, van Dijk CN (2008). The anatomy in relation to injury of the lateral collateral ligaments of the ankle: a current concepts review. Clin Anat.

[21] Campbell KJ, Michalski MP, Wilson KJ, Goldsmith MT, Wijdicks CA, LaPrade RF, Clanton TO (2014). The ligament anatomy of the deltoid complex of the ankle: a qualitative and quantitative anatomical study. J Bone Joint Surg Am.

[22] Norkus SA, Floyd RT (2001). The anatomy and mechanisms of syndesmotic ankle sprains. J Athl Train.

[23] Mahan J, Damodar D, Trapana E (2020). Achilles tendon complex: the anatomy of its insertional footprint on the calcaneus and clinical implications. J Orthop.

[24] Gorbachova T (2020). Magnetic resonance imaging of the ankle and foot. Pol J Radiol.

[25] Michels F, Pereira H, Calder J (2018). Searching for consensus in the approach to patients with chronic lateral ankle instability: ask the expert. Knee Surg Sports Traumatol Arthrosc.

[26] Halabchi F, Hassabi M (2020). Acute ankle sprain in athletes: clinical aspects and algorithmic approach. World J Orthop.

[27] Liu K, Ji X, Su P (2025). Advancements in minimally invasive treatment of deltoid ligament injuries combined with distal tibiofibular syndesmosis injuries. BMC Surg.

[28] Mollon B, Wasserstein D, Murphy GM, White LM, Theodoropoulos J (2019). High ankle sprains in professional ice hockey players: prognosis and correlation between magnetic resonance imaging patterns of injury and return to play. Orthop J Sports Med.

[29] Raikin SM, Elias I, Zoga AC, Morrison WB, Besser MP, Schweitzer ME (2007). Osteochondral lesions of the talus: localization and morphologic data from 424 patients using a novel anatomical grid scheme. Foot Ankle Int.

[30] Walther M, Gottschalk O, Madry H (2023). Etiology, classification, diagnostics, and conservative management of osteochondral lesions of the talus. 2023 recommendations of the working group “clinical tissue regeneration” of the German Society of Orthopedics and Traumatology. Cartilage.

[31] Loomer R, Fisher C, Lloyd-Smith R, Sisler J, Cooney T (1993). Osteochondral lesions of the talus. Am J Sports Med.

[32] Godoy-Santos AL, Fonseca LF, de Cesar Netto C, Giordano V, Valderrabano V, Rammelt S (2021). Ankle osteoarthritis. Rev Bras Ortop (Sao Paulo).

[33] Kloprogge SE, Katier NN, Mailuhu AK (2023). Prevalence of radiographic ankle osteoarthritis in different subgroups of patients referred for ankle radiography. Semin Arthritis Rheum.

[34] Maffulli N, Via AG, Oliva F (2015). Chronic Achilles tendon disorders: tendinopathy and chronic rupture. Clin Sports Med.

[35] Vo TP, Ho GW, Andrea J (2021). Achilles tendinopathy, a brief review and update of current literature. Curr Sports Med Rep.

